# A Complex of Marine Geophysical Methods for Studying Gas Emission Process on the Arctic Shelf

**DOI:** 10.3390/s23083872

**Published:** 2023-04-10

**Authors:** Artem A. Krylov, Roman A. Ananiev, Denis V. Chernykh, Dmitry A. Alekseev, Ermolay I. Balikhin, Nikolay N. Dmitrevsky, Mikhail A. Novikov, Elena A. Radiuk, Anna V. Domaniuk, Sergey A. Kovachev, Georgy K. Timashkevich, Vladimir N. Ivanov, Dmitry A. Ilinsky, Oleg Yu. Ganzha, Alexey Yu. Gunar, Pavel Yu. Pushkarev, Andrey V. Koshurnikov, Leopold I. Lobkovsky, Igor P. Semiletov

**Affiliations:** 1 Shirshov Institute of Oceanology, Russian Academy of Sciences, 36, Nakhimovskiy Prospekt, 117997 Moscow, Russia; 2 V.I. Il’ichev Pacific Oceanological Institute, Far Eastern Branch of the Russian Academy of Sciences, 43, Baltijskaya St., 690041 Vladivostok, Russia; 3 Tomsk State University, 36, Lenina Prospekt, 634050 Tomsk, Russia; 4 Engineering Center, Moscow Institute of Physics and Technology, 9, Institutsky Lane, 141700 Dolgoprudny, Russia; 5 Schmidt Institute of Physics of the Earth, Russian Academy of Sciences, 10, Build. 1, B. Gruzinskaya St., 123242 Moscow, Russia; 6 Lomonosov Moscow State University (MSU), 1, Leninskie Gory, 119991 Moscow, Russia

**Keywords:** marine geophysics, gas seeps, arctic seas, multibeam sonar, sub-bottom profiler, single-beam echo sounder, ocean-bottom seismographs, continuous seismoacoustic profiling, time-domain electromagnetic imaging

## Abstract

The Russian sector of the arctic shelf is the longest in the world. Quite a lot of places of massive discharge of bubble methane from the seabed into the water column and further into the atmosphere were found there. This natural phenomenon requires an extensive complex of geological, biological, geophysical, and chemical studies. This article is devoted to aspects of the use of a complex of marine geophysical equipment applied in the Russian sector of the arctic shelf for the detection and study of areas of the water and sedimentary strata with increased saturation with natural gases, as well as a description of some of the results obtained. This complex contains a single-beam scientific high-frequency echo sounder and multibeam system, a sub-bottom profiler, ocean-bottom seismographs, and equipment for continuous seismoacoustic profiling and electrical exploration. The experience of using the above equipment and the examples of the results obtained in the Laptev Sea have shown that these marine geophysical methods are effective and of particular importance for solving most problems related to the detection, mapping, quantification, and monitoring of underwater gas release from the bottom sediments of the shelf zone of the arctic seas, as well as the study of upper and deeper geological roots of gas emission and their relationship with tectonic processes. Geophysical surveys have a significant performance advantage compared to any contact methods. The large-scale application of a wide range of marine geophysical methods is essential for a comprehensive study of the geohazards of vast shelf zones, which have significant potential for economic use.

## 1. Introduction

Areas of active gas release from marine sediments are common in almost all arctic seas of the Russian sector. In the waters of the Barents and Kara Seas, signs of massive fluid discharge are described in [1,2,3,4,5]. This process is especially intensive in the seas of the Eastern Arctic and, in particular, in the Laptev Sea [6,7,8]. The Laptev Sea is the most interesting area in terms of seepage scale and seismotectonic settings, since the northern part of the Laptev Sea there is a junction of the Gakkel spreading ridge and the structures of the continental shelf [9].

The Russian sector of the arctic shelf is the longest in the world. Taking into account the fact that intensive development of offshore oil and gas fields and the Northern Sea Route has begun, as well as the development of the corresponding coastal and marine infrastructure, the study of geohazards in the northern seas of Russia is becoming a very urgent scientific and practical task. Gas streams released from the sea bottom generally weaken the structure and natural stability of bottom sediments and serve as a potential source of natural risks during the construction and operation of underwater structures, such as pipelines, oil platforms, etc. [10].

Recent studies show the mixing origin of escaping methane on the Laptev Sea shelf: with the predominance of surface biogenic origin in the innermost shelf [11] and with the predominance of deep thermogenic origin on the outer shelf of the Laptev Sea [12]. Methane, in the case when it has an endogenous, deep origin, can come from great depths along faults and reach the level of occurrence of gas hydrates, where it mixes with near-surface methane. At the same time, weak microearthquakes occurring in such areas indicate active faults. Moreover, seismotectonic events can affect the intensity of gas vents [13,14,15,16,17,18]. Thus, the processes of active gas seepage from marine sediments are of interest as an independent type of geohazards and are also a companion of active tectonic processes [19,20]. Gas seepage is accompanied by the formation of specific relief forms—pockmarks, and in areas saturated with natural gas, specific ping-like structures are formed. Pockmark fields can be used as an indicator of areas where massive fluid discharge is taking place or this process has ended in the recent past. A factor that enhances the release of gas can also be the presence of plowing places—the arctic shelf seabed is dotted with ice plow marks [21,22].

Geophysical methods are of particular importance in the comprehensive study of the process of gas seepage. Their use allows for solving a wide range of problems related to the detection, mapping, quantification, and monitoring, as well as the study of upper and deeper geological roots of gas emission and their relationship with tectonic processes. The purpose of this paper is to review a complex of marine geophysical methods used in the study of the gas seepage process in the Russian sector of the arctic seas.

## 2. Marine Geophysical Methods for Studying Gas Emission Processes

### 2.1. Hydro- and Seismoacoustic Instruments

Three acoustic instruments were used to obtain data on the structure of the uppermost sediment deposits, the seafloor morphology, and the water column above: SES-2000 Standard sub-bottom profiler (SBP), WASSP WMB-3250 multibeam echo sounder and Simrad EK15 single-beam echo sounder. During the expedition, its main application was to detect gas bubbles in the water column. High-resolution sub-bottom profiling was also used for the choice of definitive station points for deep coring.

**Figure 1:**
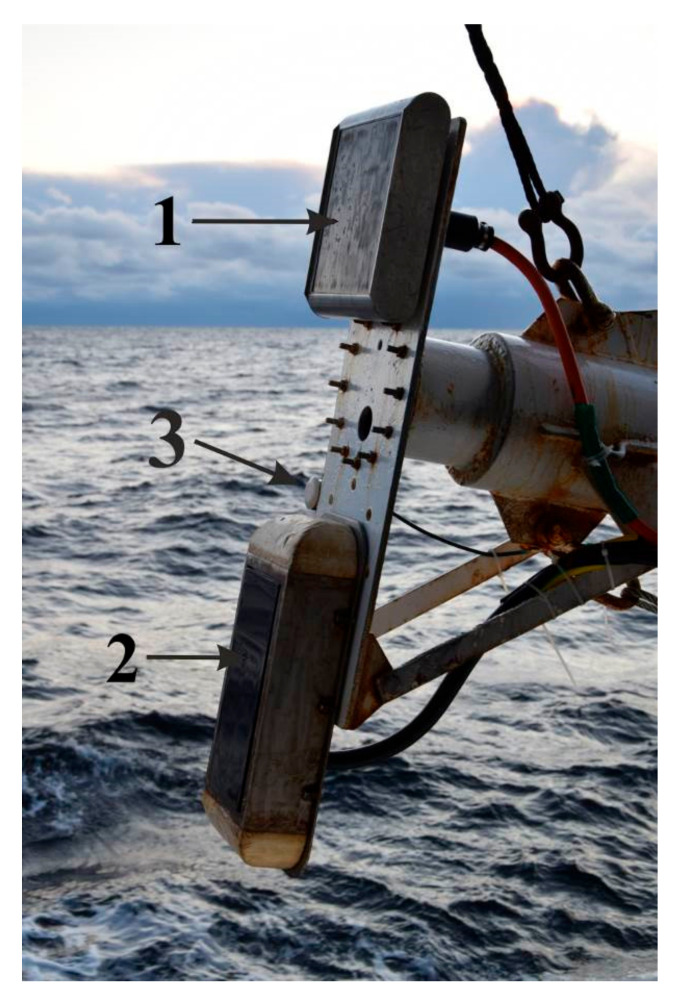
The pipe with echo sounders and SBP transducers in the marching position. 1—SES-2000 transducer, 2—WASSP WMB-3250, 3—Simrad EK-15 transducer (adopted from [10]).

The transducers of the acoustic equipment were installed on the same pipe, which made it easy to switch from transitional to working position (Figure 1). The pipe is placed on the main deck at a distance of approximately one-third of the length from the stern of the vessel. In the working position, the pipe was lowered vertically down, which made it possible to lower the converters below sea level by 4 m. The positioning system was installed on the main deck near the pipe. The antenna of a satellite compass was installed over a place of attachment of the pipe.

The travel speed was about 5 knots and was limited by the mechanical parameters of the pipe attachment to the vessel. The multibeam echo sounder antenna was lowered below the keel, which made it possible to receive bathymetric data with seafloor coverage reaching 4–5 depths.

#### 2.1.1. WASSP WMB-3250 Multibeam System

**Figure 2:**
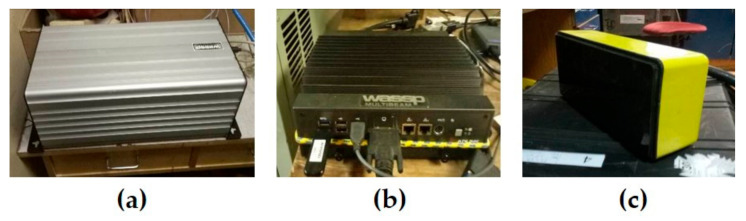
Components of the multibeam echo sounder WASSP WMB-3250: BTxR transceiver (**a**), WASSP processor (**b**), transducer (**c**).

Multibeam echo sounder WASSP WMB-3250 (manufactured by WASSP Limited, Auckland, New Zealand) is the system of bathymetry and water column data acquisition for water depths 2–200 m. The multibeam system consists of a transceiver BTxR, a WASSP processor, and a transducer (Figure 2). Real-time heading, attitude, and position are provided to the WMB-3250 by an integrated satellite compass and positioning system. The basic technical characteristics of the WASSP multibeam system are given in Table 1.

#### 2.1.2. SES-2000 Standard Sub-Bottom Profiler

**Figure 3:**
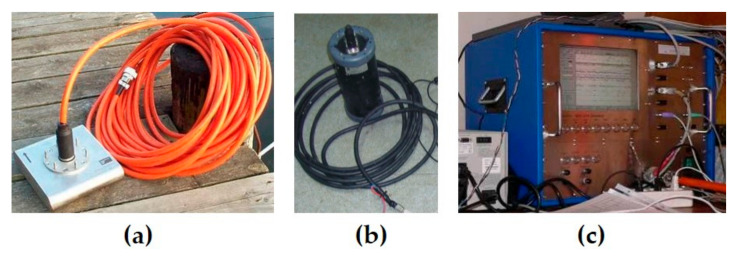
Components of the sub-bottom profiler SES-2000 Standard: SBP transducer with cable (**a**), positioning sensor (**b**), topside unit (**c**).

The SES-2000 Standard narrow-beam parametric sub-bottom profiler (manufactured by Innomar Technologie GmbH, Rostock, Germany) (Figure 3, Table 2) is a two-channel acoustic system consisting of three main elements: an SBP transducer, a topside unit, and a positioning system sensor. The transducer emits two high-power and high-frequency nonlinear signals with frequencies near 100 kHz. In accordance with the laws of nonlinear acoustics, difference-frequency signals are formed in the water column, which are then used.

The main advantage of the generated difference-frequency signals is their narrow radiation pattern, the almost complete absence of side lobes, and good penetration of the low-frequency channel into sediments. At the same time, the high-frequency channel is used as an echo sounder for bathymetric surveys. Depending on the signal frequency ratio, the low-frequency profiling pulse can have a frequency from 4 to 15 kHz.

#### 2.1.3. Simrad EK15 Single-Beam Echo Sounder

The Simrad EK15 scientific single-beam echo sounder is used to study the water column and the seabed at depths from 1 to 200 m. It is designed to provide information on volumetric sound backscatter levels from acoustic anomalies in the water column. Due to its small dimensions and low power consumption, this model allows performing research in hard-to-reach places (shallow lagoons, rivers, and lakes). The basic technical characteristics of the Simrad EK15 echo sounder are given in Table 3.

**Figure 4:**
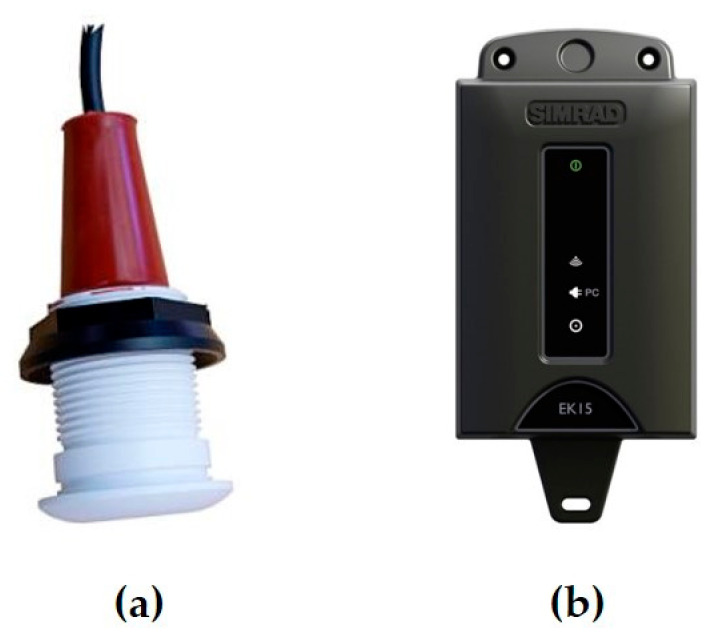
Components of the Simrad EK15 Single-Beam Echo Sounder: transducer (**a**) and transceiver unit (**b**).

This system is based on a small, sealed single-beam transceiver antenna and a data processing system (Figure 4). The operating frequency of this device is 200 kHz, which allows for getting a high-resolution in depth. Data obtained during the reception of the reflected acoustic signal is collected and accumulated using Simrad EK15 software.

#### 2.1.4. Continuous Seismic Profiling System

**Figure 5:**
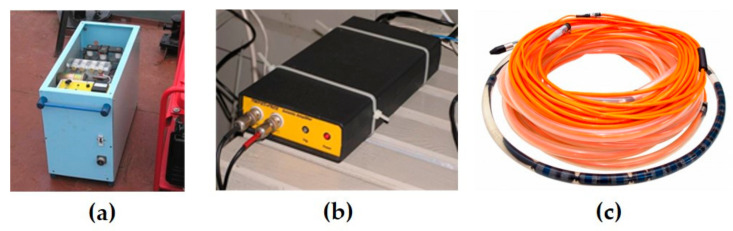
Components of the CSP system Geont-Shelf: energy source (**a**), seismic recorder (**b**), and towed streamer with sparker (**c**).

The continuous seismic profiling (CSP) system Geont-shelf includes the SPES-600 energy source, the PSA-1 seismic recorder, a towed hydrophone streamer, and a seismic source (sparker) (Figure 5). Sparker is a marine seismic source, which generates an acoustic signal by discharging an electrical pulse. The sparker produces a highly repeatable broadband signal, suitable for any type of high-resolution seismic surveys with vertical resolution up to 30 cm for geohazard assessment, detailed stratigraphic studies, etc. The frequency composition of the emitted signal can be controlled by the number of tip levels and energy per electrode involved in the pulse generation. The main technical characteristics of the CSP system Geont-Shelf are given in Table 4.

### 2.2. Time-Domain Electromagnetic (TDEM) Imaging System

**Figure 6:**
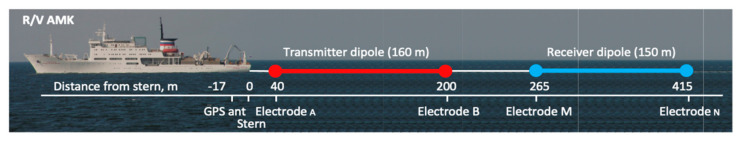
Typical configuration of the towed electrical array used in marine surveys for TDEM acquisition.

Time-domain (or Transient) electromagnetics (TDEM or TEM) is a controlled-source EM geophysical method using measured electromagnetic decay response to image the subsurface resistivity [23,24], which can be used in both onshore and marine environments [25]. The key concept of this technique implies the excitation of a primary electromagnetic field using a square wave-form current transmitted into a dipole or loop antenna, followed by measuring the secondary EM field arising in the conducting medium due to EM induction (Faraday’s Law). Depending on the receiver type, TDEM data have the form of transient response either in terms of voltage (measured by dipole) or magnetic field change rate (if measured by loop). The measured transient process, also referred to as response or decay curve, is a function of time passed after the current is cut off, and the shape of this curve plotted in log-log scale reflects the subsurface resistivity structure (the depth-resistivity profile in the simplest case of 1-D resistivity model). The time variable plays the role of a sounding parameter that controls the depth of field propagation, where earlier time corresponds to a shallower depth, while the later time corresponds to a greater depth. Offshore TDEM operations employ towed dipole-dipole arrays, in which electric current pulses are transmitted via the line, providing galvanic contact between the seawater and the electrodes placed at the ends of the source dipole. The received signal is a voltage measured between two other electrodes, towed behind the transmitter line. Both lines can be submerged or float on the water’s surface. The geometry of the particular array used in the Laptev Sea survey is shown in Figure 6.

In terms of electrical hardware, the acquisition system includes a five-channel receiver (Tells-3E) with 32-bit ADC that is employed for voltage recording, both from the towed receiver dipole and transmitter output and Forpost 105 kW transmitter providing a maximum voltage of 260 V and square waveform current up to 400 A. Steel and brass electrodes were used to provide galvanic contact between the dipoles and seawater.

During the survey, the voltages are recorded in continuous TDEM acquisition mode, with individual responses (stations) collected every 4 s (having 1 ms sampling rate) within a 2 s interval following another 2 s current transmission phase. Depending on the vessel speed (4–10 knots), the station spacing varies from 13 to 50 m. Practically, the transmitted current value (pulse amplitude) is between 170 and 200 A and is continuously recorded along with voltage measurements with the same sampling rate. The typical dipole array is shown in Figure 6, although particular geometrical features may vary depending on the survey design.

### 2.3. Ocean-Bottom Seismographs (OBS)

**Figure 7:**
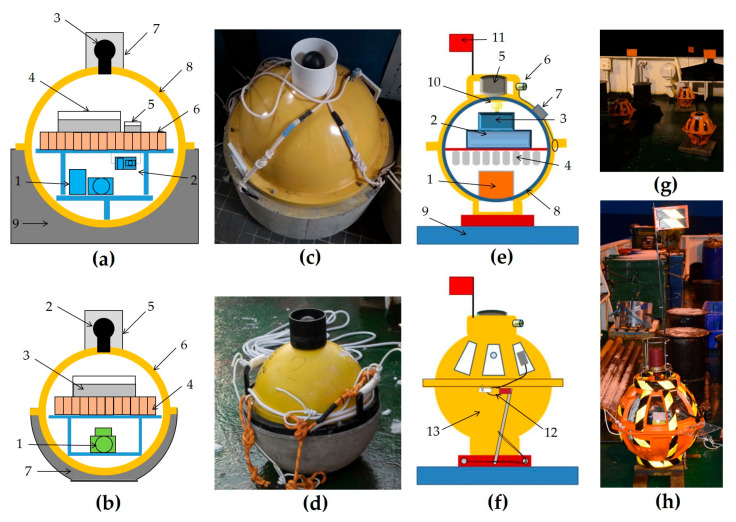
(**a**) The design of the MPSSR ocean-bottom seismograph: (1) three-component broadband seismometer CME-4311, (2) three-component short-period seismometer (SV-10 and SH-10) placed in gimbal, (3) hydrophone 5007 m, (4) recorder URS-S, (5) digital compass module, (6) battery block, (7) protective half-cover for hydrophone, (8) duralumin sphere, and (9) concrete ballast; (**b**) the design of the Typhoon ocean-bottom seismograph: (1) three-component short-period seismometer CME-3311, (2) hydrophone 5007 m, (3) recorder URS-S, (4) battery block, (5) protective half-cover for hydrophone, (6) duralumin sphere, and (7) concrete ballast; (**c**) MPSSR external view on the R/V Akademik Mstislav Keldysh, autumn 2018; (**d**) Typhoon external view on the R/V Akademik Mstislav Keldysh, autumn 2019; (**e**,**f**) the design of the GNS and GNS-C ocean-bottom seismographs: (1) Three-component broadband seismometer CME-4111, (2) recorder GNS, (3) acoustic modem, (4) batteries block, (5) acoustic hydrophone, (6) seismic hydrophone, (7) penetrator, (8) glass spherical housing with diameter 430 mm, (9) anchor, (10) lamp, (11) flag, (12) anchor release, (13) plastic case. (**g**) GNS, and (**h**) GNS-C external view on the R/V Akademik Mstislav Keldysh, autumn 2018 and 2019 correspondingly.

The two OBS models MPSSR (abbreviation from Russian: sea bottom station for seismoacoustic exploration) and Typhoon developed by the Shirshov Institute of Oceanology, Russian Academy of Sciences (IO RAS) are suitable for a wide range of tasks, including seismological monitoring, active and passive seismics, and high-resolution seismoacoustic investigations. The design of the MPSSR and Typhoon stations and their external view are presented in Figure 7a–d. The basic parameters are presented in Table 5. Both models are equipped with three-component MET seismometers: CME-4311 type (0.0167–50 Hz) in the MPSSR station and CME-3311 type (1–50 Hz) in the Typhoon station (R-sensors, Moscow, Russia [26]). The hydrophone 5007 m used has a frequency range of 0.04–2500 Hz. The MPSSR is also equipped with SV-10 and two SH-10 classic electromechanical geophones, with a frequency range of 10–250 Hz (analogy of GS-20DX), placed in a gimbal [20,27].

The MPSSR and Typhoon OBSs housing is rigidly attached to the concrete ballast to improve the traction on the seabed. The current equipment is not self-pop-up and the deployment in shallow waters is conducted with the use of external acoustic release and buoys. The deployment scheme allows trawling of a rope laid on the seabed between the OBS and the ballast-buoy system if the acoustic release does not work after long-term operation. Thus, the current equipment and the deployment scheme imply work on the shelf at depths of no more than 100 m [20,27].

Unlike the MPSSR and Typhoon models described above, the GNS and GNS-C models are used for scientific applications of the IO RAS in deep waters (up to 6000 m) [19,20]. It was developed by the IP Ilinskiy A.D. and IO RAS. The GNS employs the SM-6 electro-dynamic sensors, and GNS-C employs 120 s MET sensor CME-4111 (R-sensors, Russia, [26]). These OBSs are self-pop-up with water depths ranging up to 6000 m and are suitable for studying deep structures, down to the middle mantle by active and passive seismic methods and also for earthquake seismology. The design of the GNS and GNS-C models differ mainly by size, and their external views are presented in Figure 7e,f. The general characteristics are shown in Table 6.

## 3. Results of Research in the Laptev Sea

### 3.1. Detection and Quantification of Gas Flares

**Figure 8:**
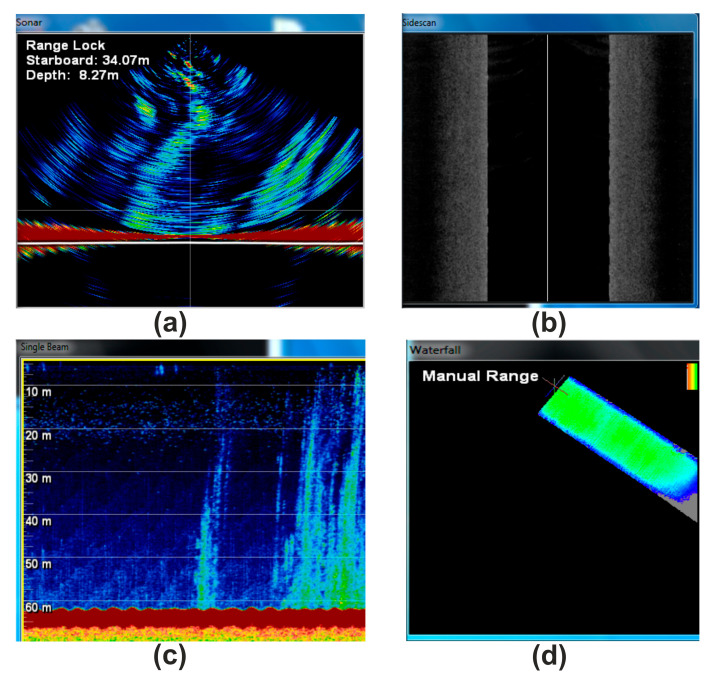
Screenshot of WASSP operating software demonstrating the available view types: water column sonar data (**a**), backscatter data (**b**), single beam data (**c**), and 3-D waterfall view of multibeam data (**d**). The record made at a time when a vessel passed through a gas flare in the Laptev Sea.

**Figure 9:**
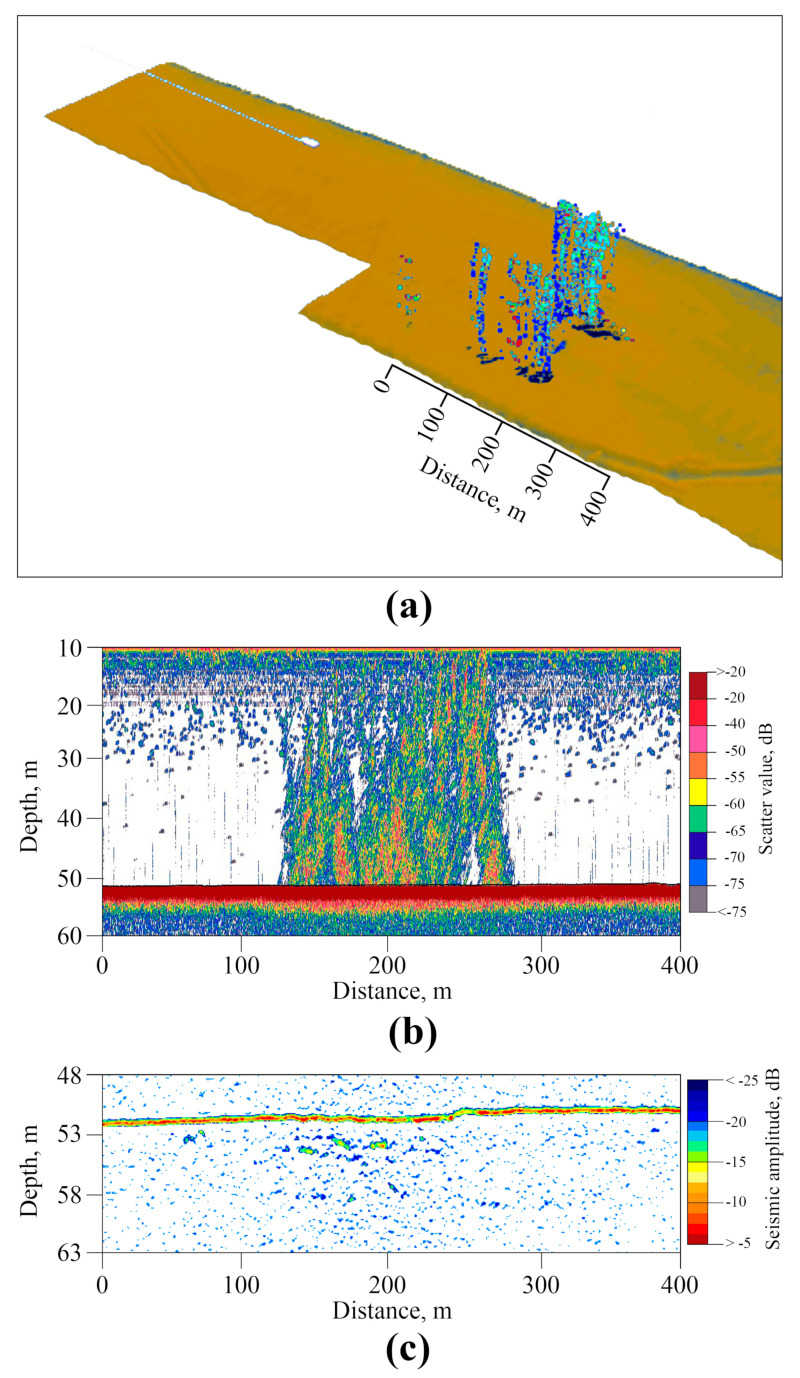
Multibeam (**a**), single beam (**b**), and SBP data (**c**) were obtained in the Laptev Sea from the same area.

The WASSP WMB-3250 system allowed the simultaneous acquisition of bathymetric, sonar, stacked beam image, and backscatter data (Figure 8). Gas bubbles in the water column have been detected in many areas of the Laptev Sea using this multibeam system. Figure 9 shows multibeam, single beam, and sub-bottom profiler data from the same area. The simultaneous use of the single-beam and the multibeam system made it possible to confidently identify areas of increased sound dispersion associated specifically with the underwater release of natural gas.

**Figure 10:**
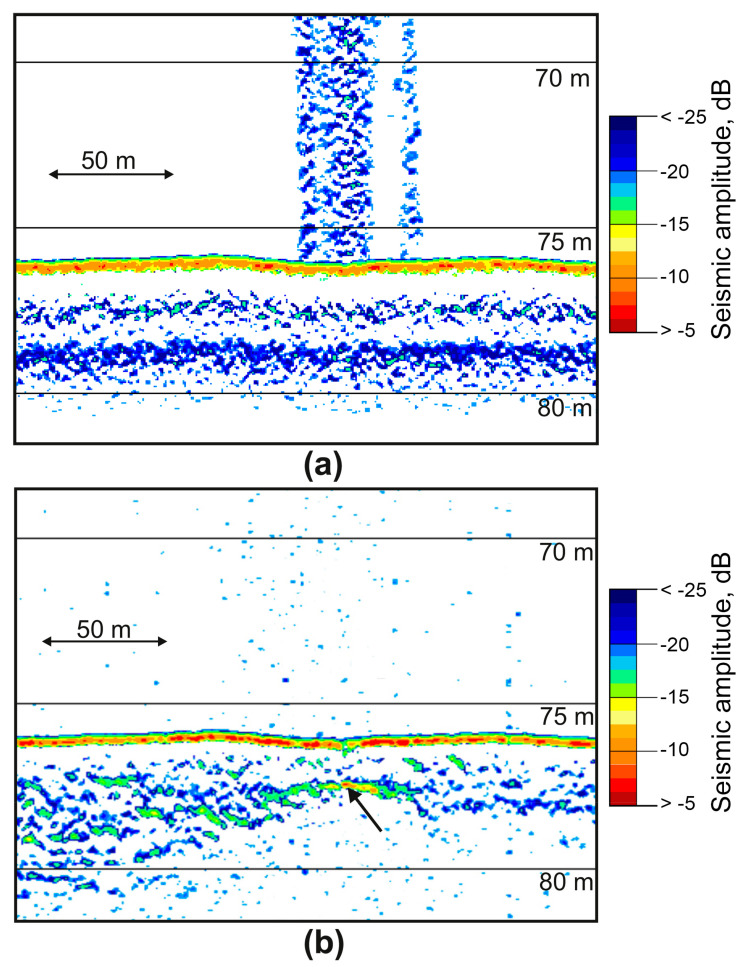
Simultaneous records of gas anomalies were obtained in the Laptev Sea using the high frequency (**a**) and low frequency (**b**) channels of SES-2000 Standard. The black arrow indicates enhanced reflection.

**Figure 11:**
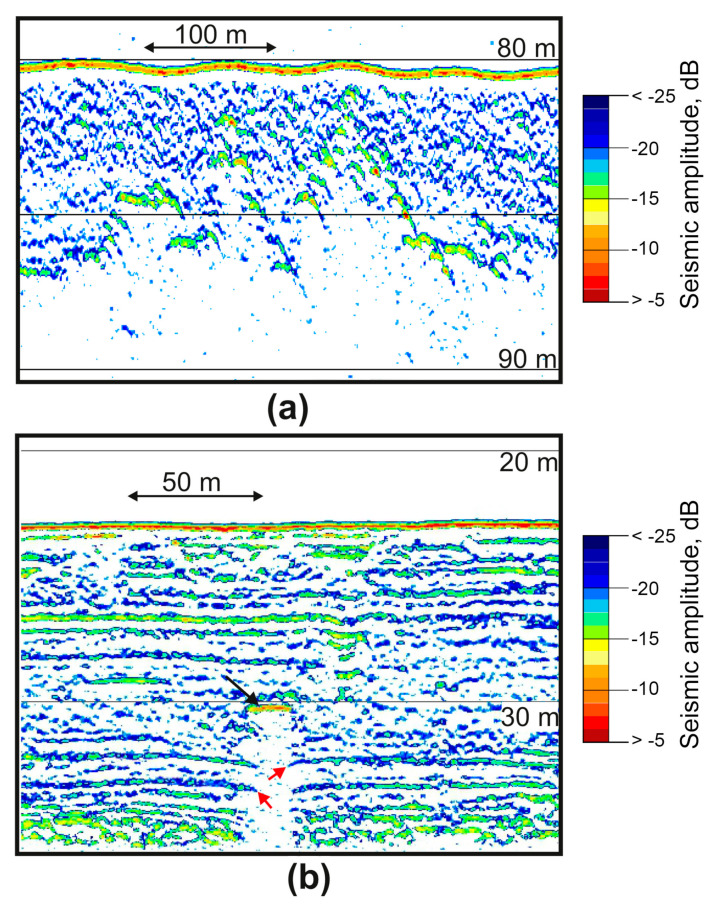
Fragments of seismic sections with signs of anomalous gas saturation of sediments based on SES-2000 data obtained in the Laptev Sea: the appearance of many diffracted waves (**a**), enhanced reflection amplitudes (black arrow) and pull-down reflectors (red arrows) (**b**).

We used the SES-2000 profiler to confidently identify the supposed zones of anomalous gas saturation of sediments, the main signs of which on seismic sections were a sharp increase in the amplitude of reflections (Figure 10b); the appearance of many diffracted waves (Figure 11a); dome- and cone-shaped vertically oriented areas of acoustically transparent or chaotic recording (Figure 11b).

High-amplitude enhanced reflection was identified at a depth of 7 m from the bottom in the central part of the seismic section in Figure 11b (black arrow). It is accompanied by an underlying vertical area of no reflections—the acoustic blanking zone. Such screening of the underlying section is caused by increased absorption of vibrational energy in gas-saturated deposits, especially in the high-frequency part of the spectrum.

Another detected effect is a delay in the registration of the underlying reflections located below the units of gas-charged sediments, causing their false deflection (pull-down reflectors) (Figure 11b). This phenomenon is caused by a decrease in the wave velocity in gas-saturated deposits [28]. This effect gives the impression of sediment subsidence, but this is just an artifact. In Figure 11b, this effect is seen in the immediate vicinity of the boundaries of the acoustic blanking zone under the amplitude anomaly (red arrows).

**Figure 12:**
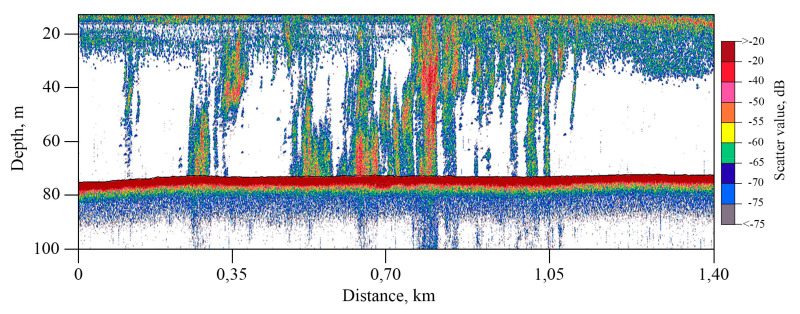
Fragment of a profile with a recording of the high-frequency Simrad EK15 single-beam echo sounder obtained in the Laptev Sea.

The acoustic image of a single jet obtained by the Simrad EK15 single-beam echo sounder has the form of a vertically oriented zone of continuous illumination with a horizontal dimension close to the real horizontal dimensions of the gas jet at the bottom (Figure 12). At the same time, the quality of visual reproduction of bubbles on echograms significantly depends on a number of factors: the speed of the ship, the presence of acoustic interference, the direction of underwater currents that affect the trajectory of the rising bubbles, and the direction from which the gas flow is irradiated by the sonar device. This single-beam echo sounder makes it possible to use the data obtained to quantify the gas flow in the areas of its bubble discharge.

### 3.2. Investigation of Geological Roots of Gas Flares in Upper Part of Soil Profile

**Figure 13:**
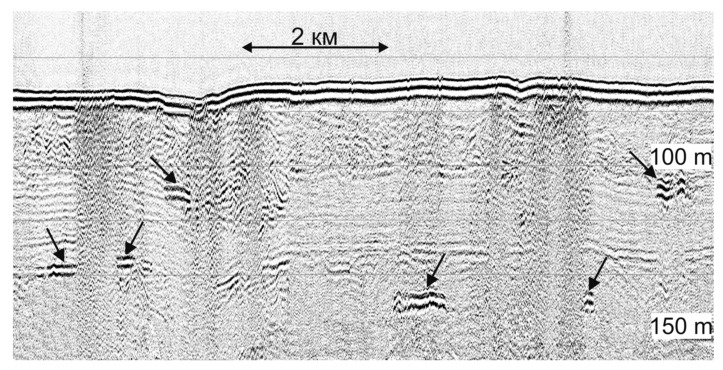
Fragment of the regional profile obtained by CSP system Geont-Shelf in the Laptev Sea. Gas-saturated layers are indicated by arrows.

Due to the high gas content in the bottom sediments, the fine structure of the upper part of the soil profile could not always be traced in seismic sections. Vertical zones of correlation disturbance and shadow zones caused by increased seismoacoustic energy absorption in the near bottom layers are visible on the CSP profile (Figure 13). In addition, the section clearly distinguishes some areas of a sharp increase in the amplitudes of reflections at the levels of 105, 130, and 145 m below the water level, interpreted as gas-saturated layers, they are shown by arrows in Figure 13. According to these data, it can be argued that the vertical migration of gas to the surface, which coincides with the places of underwater gas discharge according to echo-sounding data, occurs from a depth of at least 65 m below the bottom surface. Despite the low-frequency spectrum of the CSP system signals, the gas escape from the seabed to the water column shown in Figure 13 is quite noticeable, which may indicate a very powerful and concentrated bubble flow.

**Figure 14:**
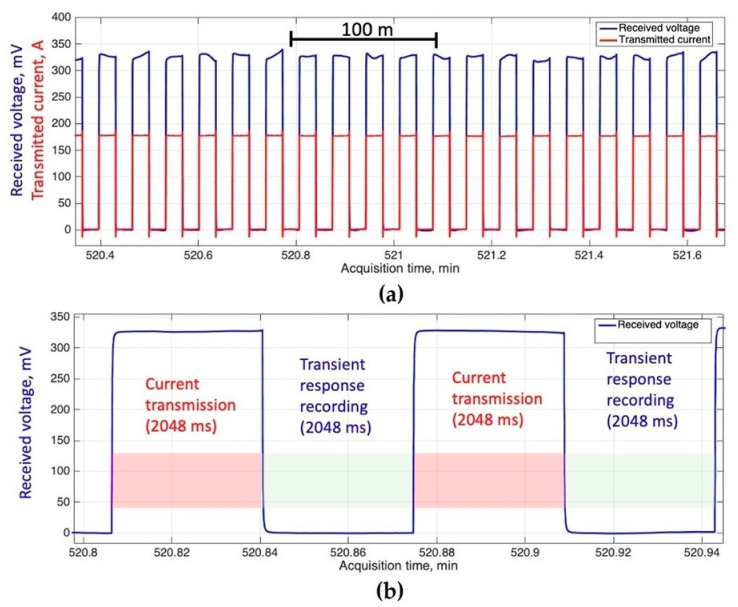
Example of TDEM time series recorded in the southern part of the Laptev Sea. The received voltage is shown in blue, while the transmitted current is in red. Panel (**a**) shows a 1.3 s interval with individual current pulses becoming visible; panel (**b**) provides a close-up of two sequential pulses/responses.

An example of transient electromagnetics time series recorded in the southern part of the Laptev Sea is shown in Figure 14. These time series are segments of a continuous sequence of 2 s current pulses injected into water by the transmitter dipole along with voltages measured by the receiver dipole, with each pulse corresponding to a separate station along the vessel track. These form specific time series patterns enabling us to estimate data quality and characterize bias. Figure 14a shows some 20 sequential stations, while Figure 14b indicates the transient decay (linear time scale is used) observed after the current cut-off for two sequential current pulses (stations).

**Figure 15:**
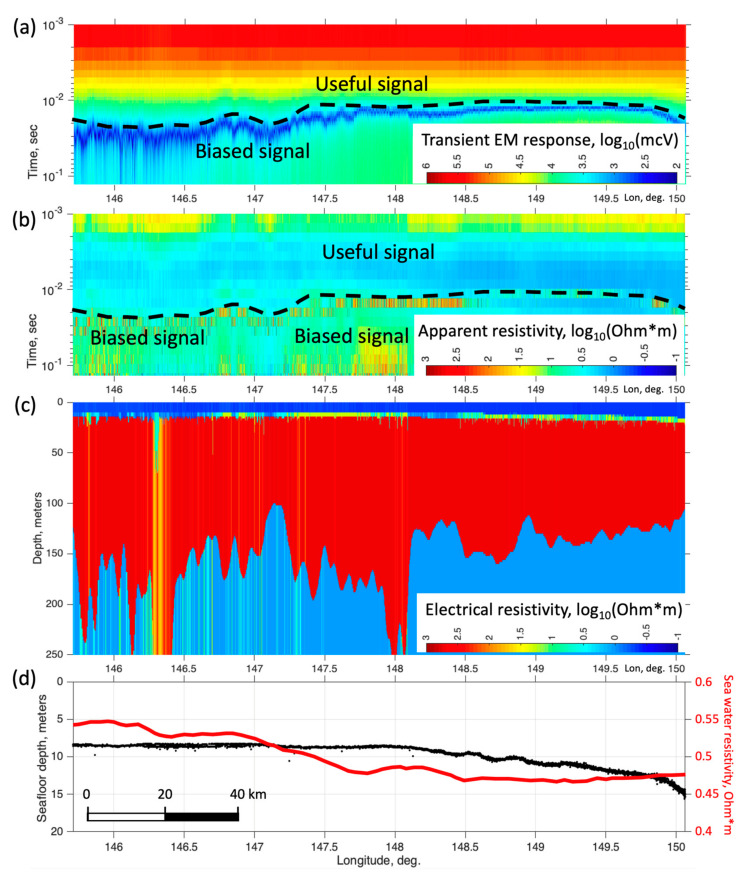
Examples of TDEM data recorded in the Laptev Sea southeast of Bolshoy Lyakhovsky Island. Raw decay voltage and processed apparent resistivity pseudosections (logarithmic color scale) are presented in panels (**a**,**b**), respectively; the dashed line corresponds to the transient time where responses start to exhibit unrealistically-high de-cay rate caused by low-frequency noise, and marks the boundary between the useful signal at earlier times and the distortion-affected region; panel (**c**) shows inverted resistivity model, with the dark-red region being the permafrost layer, and (**d**) indicates the seafloor depth (black) and surface water resistivity (red) along the line track.

An example of TDEM response data measured along a some-180 km line in the southeastern part of the Laptev Sea is shown in Figure 15 in the form of raw signal (Figure 15a) and filtered apparent resistivity (Figure 15b) pseudosections. Vertical axes represent the time after current cut off, and colormap corresponds to the log10 magnitudes, covering a wide dynamic range of the raw decay voltages, starting at around 1 V at early times and decaying rapidly down to a few mcV at late times (around 4 orders of magnitude). However, the late-time portion of the response is commonly affected by low-frequency distortions with a mean magnitude of around a few mV. The black dashed line marks the boundary between accurate and biased portions of the response, where the lower noisy region is characterized by rapid signal decay, which cannot be attributed to any variations in the sub-bottom resistivity structure. Although the pseudosections look fairly uniform within an early-time region (1–30 ms), some smooth lateral variations still can be seen, specifically, in the filtered apparent resistivity pattern (Figure 15b).

Figure 15c shows the main result of TDEM acquisition, an inverted resistivity model, imaging the subsurface structure in terms of electrical resistivity. The model was inverted from filtered apparent resistivity data, for which we applied the MATLAB-based TEM1DInv code employing Optimization Toolbox functionality. A nonlinear inversion procedure converts the apparent resistivity response at each station into the 1-dimensional (horizontally-layered) resistivity model beneath it by minimizing the objective function (data misfit) defined as the normalized sum of the squared differences of the logarithms of apparent resistivities $\rho_{a}\left(t\right)$. Summation is conducted over the time samples on a logarithmic grid:

(1) $$\Phi \left(x\right)=\sum_{1}^{Nt}\frac{1}{\delta \left(t_{i}\right)}{\left(log_{10}\rho_{a}^{obs}\left(x,\;t_{i}\right)-log_{10}\rho_{a}^{mod}\left(x,\;t_{i},\;m\left(x\right)\right)\right)}^{2},$$

where *t_i_* denotes time samples with logarithmic spacing, *N_t_* is the number of samples in a given response curve; $\rho_{a}^{input}\left(x,\;t_{i}\right)$ is input data; $\delta \left(t_{i}\right)$ characterize data errors (set to 1 in our synthetic experiment); $\rho_{a}^{mod}\left(x,\;t_{i},\;m\left(x\right)\right)$ is a modeled response, calculated from the *N*-layer resistivity model **m** referenced to station *x*:

(2) $$m\left(x\right)=\left\{\begin{array}{c} log_{10}\rho_{j}\\ log_{10}h_{j} \end{array}\right.,j=1,\ldots N$$

Thus, **m** is a vector composed of logarithmic resistivities $\rho_{j}$ and thicknesses $h_{j}$. Hence $\rho_{1}$ and $h_{1}$ correspond to the water layer, whose thickness is known and resistivity is believed to vary within a relatively narrow range (Table 7). Misfit minimum search is carried out with a modification of the gradient descent method, using the functionality of MATLAB Optimization Toolbox. Inversion is constrained inversion by specifying the bounds for each constituent of model vector **m**, representing a four-layer resistivity structure. These are chosen based on available data and estimates (Table 7) [29,30,31].

An important aspect of inversion stabilization (regularization) is that seawater depth $h_{1}$ is constrained within a 20 percent margin of the known bathymetry data, estimated from sonar data. This reduces the number of parameters being recovered and greatly facilitates inversion stability.

The code requires the initial guess (starting) model to be specified, and the profile data inversion runs successively in a way, that the final model for the current station is used as the initial one for the next station along the line. The resistivity of layer 1 (seawater) is searched within 20% bounds of the initial value, calculated at each station from salinity data assuming the Practical Salinity 1978 model [32]. Furthermore, to provide lateral smoothness of the resulting model, all parameters at each given station are constrained within a 20% margin of those at the previous station in line. The initial guess for the first station in a line is specified according to priory data and sensitivity tests. Thus, to ensure the model’s stability, the inversion code is strongly dependent on both bathymetry and salinity data (Figure 15d) measured by separate systems.

### 3.3. Investigation of Deep Roots of Gas Flares and Their Relation to Tectonic Processes

**Figure 16:**
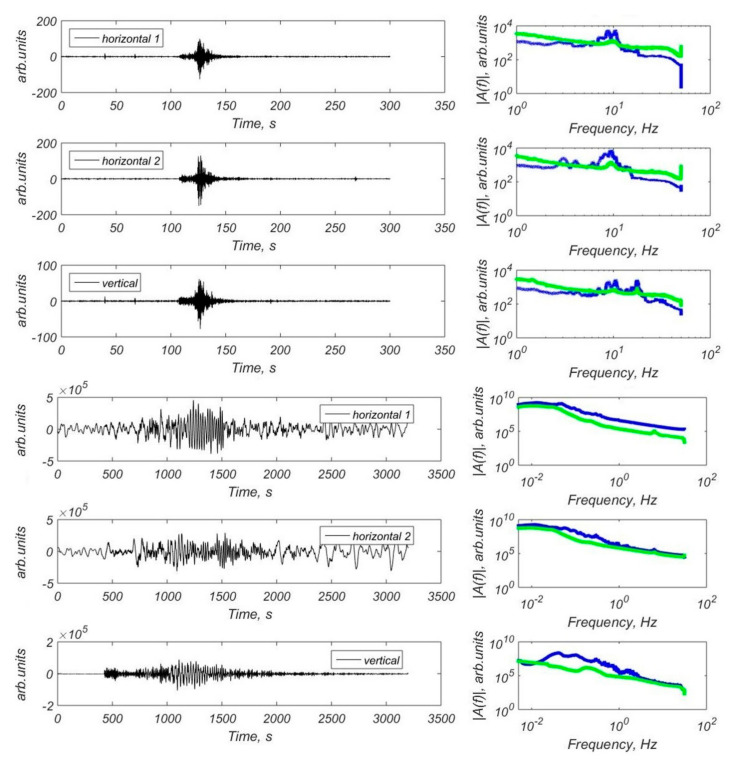
Examples of waveforms and Fourier spectra recorded by OBSs in the Laptev Sea (modified after [20]). Three upper panels: waveforms (bandpass filter 2–45 Hz applied) and the FFT spectra of the local microearthquake with P-arrival at 11 November 201901:58:31 UTC, obtained by the three-component CME-4311 channels of the MPSSR station; Three lower panels: waveforms and FFT spectra of the earthquake with M = 7.1 that occurred in Alaska (30 November 2018 17:29:29 UTC) [38] obtained by the three-component CME-4111 channels of the GNS-C station. Blue line—the spectra of the earthquake, green line—the spectra of the seismic noise preceding P-wave arrival.

In a series of scientific cruises, we obtained a significant number of signals from local and remote earthquakes in the Laptev Sea recorded by all the OBS models described above [19,20,27,33,34,35,36,37]. Examples of waveforms and Fourier spectra are shown in Figure 16.

**Figure 17:**
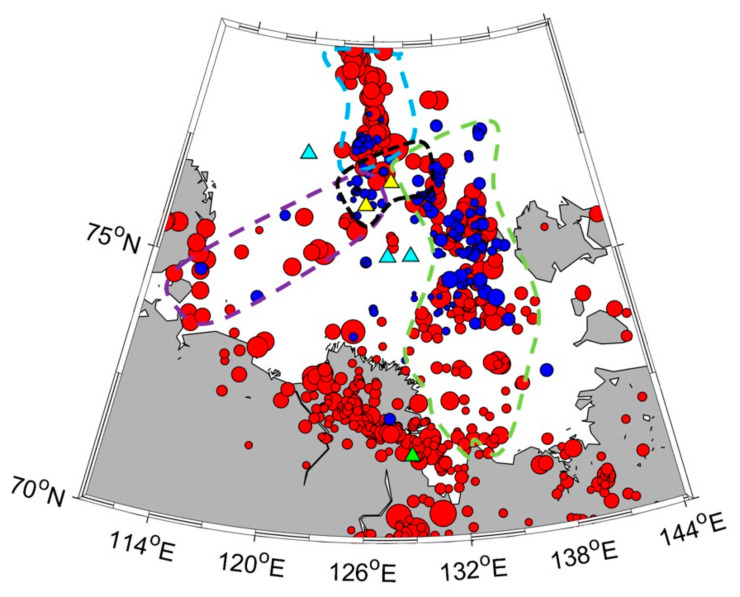
The distribution of the earthquake epicenters in the Laptev Sea region: red circles—the joint regional catalog by ISC [39], USGS [38] and “Earthquakes of Russia” database [40], blue circles—the catalog of the events that were most clearly recorded by the OBSs (several months during 2018–2020 [20,37]). Location of the OBSs that worked for several months and were successfully dismantled: cyan triangles—in the 2018–2019 season (two MPSSR on the shelf and one GNS-C on the slope); yellow triangles—in the season 2019–2020 (one MPSSR and one Typhoon); green triangle—permanent on-land broadband seismic station of the Geophysical Survey of the Russian Academy of Sciences in the village of Tiksi). The blue dashed line outlines the seismicity belts associated with the Gakkel spreading ridge; the green dashed line—with the East Laptev province of horsts and grabens; the purple dashed line—with the southwestern segment of the Khatango-Lomonosov fault zone; the black dashed line—the area of concentration of gas flares.

Figure 17 shows the distribution of the earthquake epicenters in the Laptev Sea region obtained from the joint regional catalog (ISC [39], USGS [38], “Earthquakes of Russia” database [40]) and the catalog of the events that were most clearly recorded by the OBS (several months during 2018–2020) [20,37], as well as the location of the seismic stations and the area of concentration of gas flares on the outer edge of the continental shelf [6]. The coordinates of the epicenters of recorded earthquakes were determined by a combination of the multiple-station circle method and the single-station method. It can be seen that this area of concentration of gas flares is located at the junction of three seismicity belts associated with the Gakkel spreading ridge, the East Laptev province of horsts and grabens, and the southwestern segment of the Khatango-Lomonosov fault zone.

## 4. Discussion

Various types of hydroacoustic devices are used to detect and map gas flares in many areas of the World Ocean, including single-beam and multibeam echo sounders, split-beam echo sounders, side-scan sonar, and other sonars, including passive ones [41,42,43,44,45]. Our studies have confirmed that the most informative from the point of view of direct registration of gas flares are high-frequency echo sounding performed, for example, by such devices as the Simrad EK15 single-beam echo sounder, which makes it possible to identify almost all forms of gas release—from single small sources to continuous gas flares hundreds of meters or more in length. An important advantage of single-beam echo sounding systems is the possibility of using the data obtained to quantify the gas flow in the areas of its bubble discharge. Conventional remote flow measurement techniques are based on estimating the volume of bubble gas from the sound backscatter level using additional data from other methods [10,44,46].

In the context of studies of methane bubble discharge areas using single-beam echo sounders, an expected development is to move from the estimation of the flow of methane into the water to estimates of the flow to the seawater-atmosphere interface. To do this, it is necessary to integrate the coefficients that take into account gas exchange between these bubbles and the liquid column through which they float or develop new methods that take into account this process in the available methods for estimating the amount of methane carried by floating bubbles [6,44,47,48,49].

In the seas of the Russian Arctic, gas flares have been found on the continental slope at depths up to 400 m or more [6,50]. Floating bubbles make it possible to avoid the oxidation of the methane carried by them in the sulfate reduction layer [11,51]. Due to the underestimation of this factor, the role of the World Ocean as a source of methane is greatly underestimated (5–10 million tons per year). It is significantly lower than the current estimate of its flow, made only for the shelf of the Eastern Arctic seas and amounting to 17 million tons per year [6,52]. At the same time, it was found that the amount of bubble transfer from the bottom sediments of the East Siberian Seas shelf into the water column, depending on the state of the underwater permafrost, changes by five orders of magnitude from 0.001 to 1000 g per square meter per day [49].

The use of single-beam echo sounders for spatial mapping of the position of gas flares, although it can detect signs of gas emissions, is limited by the fact that the system is single-channel, and a frequent grid of lines is required to provide an accurate areal mapping of gas outlets. To quickly obtain a picture of the spatial distribution of gas flares, it is more effective to use the WASSP WMB-3250 multibeam echo sounder. In addition, the bathymetric area survey makes it possible to detect pockmarks—landforms directly related to the zones of underwater natural gas discharge, as well as some other associated complications of the seabed topography. Another important option of the WASSP WMB-3250 is the possibility of real-time 3D data visualization (Figure 9a). Recently, a method using calibration measurements of the dependence of the vertical volumetric flow on the acoustic backscattering cross-section of backscattering of popping bubbles has been used, which makes it possible to perform a quantitative assessment using the acoustic data of multibeam echo sounders [10,43].

In addition to the direct registration of gas emanating from the bottom, registration of places where such emission is potentially possible and most probable is of great interest. For these purposes, the most effective is the use of seismoacoustic equipment, such as the SES-2000 sub-bottom profiler. The ability to use low-frequency and high-frequency channels together is an advantage of the SES-2000 system. Simultaneous recording of two channels allows real-time detection of areas of gas release, as well as associated areas of increased gas saturation in the sedimentary stratum (Figure 10). In order to investigate the geological roots of gas flares in the upper part of the soil profile at sub-bottom depths up to several hundred meters the CSP data is to use. Vertical zones of correlation disturbance in the upper part of the soil profile are due to the high gas content in the bottom sediments while the sharp increase in the amplitudes of reflections can be interpreted as gas-saturated layers [10]. The sampling and analysis of the gas content of cores at the locations of gas anomalies confirmed the interpretation of such sections as gas-saturated zones. The concentration of methane in the core at the depth of the acoustic anomaly increased sharply, in some cases by two orders of magnitude [7].

In the arctic seas, which are characterized by the presence of underwater ice-boned permafrost (IBP), the concentration of gas flares is associated with the state of IBP [6,7,8]. Due to the significant effect of freezing/thawing transition on the electrical resistivity of fluid-saturated sedimentary rock, electrical and electromagnetic methods are particularly beneficial for IBP imaging, and have found extensive application in permafrost-related studies [31]. A large part of resistivity-based studies utilizes the electrical resistivity tomography (ERT) method to image the seafloor rock structure in shallow-water coastal arctic environments [53,54,55]. However, being efficient in shallow-water (1–3 m) coastal environments, ERT has essential limitations in terms of depth of investigation (DOI), barely exceeding 30–40 m [56]. Deeper-water setting requires the application of the technique providing higher DOI, and TDEM is one of such methods [23,24]. Marine TDEM modification is also an efficient technique [57,58,59,60] that has been widely used in recent years in the studies of the sedimentary strata and permafrost rock in the shallow water of the arctic shelf [7,31]. The basic limitations of this technique are associated with the need for accurate measuring the late-time portion of the transient response curve, where the signal is usually extremely low and biased due to rapid decay, thus restricting the imaging depth (depth of investigation, DOI) by around 150–250 m, depending on seawater depth and conductivity.

The resistivity model presented in Figure 15c is a characteristic example of a subsurface structure image, with the dark-red region being the permafrost layer, revealing substantially lower thicknesses (within 100–200 m) compared to existing heat transfer modeling-based estimates for this area [61]. Thus, TDEM acquisition/inversion technique yields large amounts of high-resolution data enabling in-situ probing of permafrost thickness and lateral distribution aiming at a better understanding of the extent of the IBP formation in the study area.

A huge amount of TDEM time series data, counting over 150 thousand stations has been collected during the recent surveys. These require thorough analysis, processing, and inversion which are expected to provide detailed estimates of permafrost layer structure and properties in the survey area. Given the substantial effect of low-frequency noise on the measured voltage data, future improvement of the TDEM system in use might include pseudorandom current waveforms used in combination with appropriate data-processing codes [58].

During the OBS deployments in the Laptev Sea, we obtained a significant number of short-period signals from local microearthquakes and long-period signals from remote large earthquakes, both body waves and surface waves within a broad frequency range. The main characteristics of the broadband MET sensors, such as permissible installation angles, temperature range, sensitivity, dynamic range, and frequency band, appeared to be suitable for obtaining the OBS records under the arctic conditions to solve the wide range of seismological problems [20]. The data obtained in a series of scientific cruises, show that the main area of concentration of gas flares in the Laptev Sea is located at the junction of three seismicity belts associated with the Gakkel ridge, the East Laptev province of Horsts and Grabens, and the southwestern segment of the Khatango-Lomonosov fault zone. This can be explained by the fact that it is a junction of the Gakkel spreading ridge and the structures of the continental shelf, which leads to the formation of a vast deformed and disturbed area of the earth’s crust. The extensive network of deep and surface faults formed as a result of strong deformations can serve as ways for supplying deep gas to the upper layers of the geological section. The predominance of deep thermogenic origin of escaping gas on the outer shelf of the Laptev Sea [12] can serve as an argument in favor of the described mechanism.

The area of the junction of the shelf structures of the Laptev Sea with the Gakkel Ridge is extremely interesting from the geodynamic and seismological points of view as a transition from the spreading zone to the continental margin. Further study of this area is needed using a more extensive network of ocean-bottom seismographs both for a detailed description of the seismic regime and for the application of seismic tomography methods.

## 5. Conclusions

The massive release of bubble gas from marine sediments in the arctic seas of Russia is a widespread and complex process that requires a detailed multidisciplinary study. The experience of using the above equipment and the examples of the results obtained in the Laptev Sea have shown that these marine geophysical methods are effective and of particular importance for solving most problems related to the detection, mapping, quantification, and monitoring of underwater gas release from the bottom sediments of the shelf zone of the arctic seas, as well as the study of upper and deeper geological roots of gas emission and their relationship with tectonic processes.

In addition, geophysical surveys have a significant performance advantage compared to any contact methods. The large-scale application of a wide range of marine geophysical methods is essential for a comprehensive study of the geohazards of vast shelf zones, which have significant potential for economic use.

## Figures and Tables

**Table 1:** Main technical characteristics of the WASSP WMB-3250 multibeam system.

Parameter	Value
Frequency	160 kHz
Beam width	224 beams equidistant spacing over 120° port/starboard swath
Seafloor coverage	Up to 3.4 × depth
TX rate	Automatic ping rate, determined by depth. Max ping rate 40 Hz.
Output power	Up to 1 kW
Depth range	2–200 m
Depth resolution	7.5 cm
Transducer dimensions	33–17–10 cm

**Table 2:** Main technical characteristics of the sub-bottom profiler SES-2000 Standard.

Parameter	Value
Primary frequencies	approx. 100 kHz (band 95–110 kHz)
Secondary low frequencies	4–15 kHz
Pulse width	0.07–0.8 ms
Pulse rate	Up to 30/s
Power consumption	Up to 1 KW
Beam width	±1.8°
Water depth range	1–500 m
Penetration	Up to 50 m
Layer resolution	Up to 5 cm

**Table 3:** Main technical characteristics of the Simrad EK15 Single-Beam Echo Sounder.

Parameter	Value
Operational frequency	200 kHz
Typical depth range	200 m
Ping rate	up to 40 Hz
Pulse durations	80 to 1240 μs
Data rate	1.6 Mbps
Maximum number in use	15
Output power	45 W
Raw data	EK60 format
Maximum installation depth	600 m
Beamwidth	26°

**Table 4:** Main technical characteristics of the continuous seismic profiling system.

Parameter	Value
Seismic recorder PSA-1	
Frequency range	60–1200 Hz
Dynamic range	120 dB
Gain ratio	1 to 1000
SPES-600 energy source	
Maximum voltage	5 kV
Operating energy	5–600 J
Towed streamer	
Number of channels	1 to 32
Interval between channels	2 m

**Table 5:** Main technical characteristics of the non-self-popup OBSs.

Parameter	Value
MPSSR	
Sensors	three-component seismometer CME-3311, three-component geophone SH/SV-10, hydrophone 5007 m
Frequency Band (CME-4311)	0.0167–50 Hz
Sensitivity (CME-4311)	2000 V/(m/s)
Frequency Band (SH/SV-10)	10–250 Hz
Sensitivity (SH/SV-10)	28 V/(m/s)
Frequency Band (5007 m)	0.04–2500 Hz
Sensitivity (5007 m)	7.2 ± 0.5 mV/Pa
Maximum depth	3000 m
Sample rates, Hz	20, 25, 40, 50, 80, 100, 160, 200, 400, 800
Time synchronization	GPS interface
Temperature stability of the quartz generator	±5 × 10^−9^
Memory	SD card up to 64 Gb
Typhoon	
Sensors	three-component seismometer CME-3311, hydrophone 5007 m
Frequency Band (CME-3311)	1–50 Hz
Sensitivity (CME-3311)	2000 V/(m/s)
Frequency Band (5007 m)	0.04–2500 Hz
Sensitivity (5007 m)	7.2 ± 0.5 mV/Pa
Maximum depth	2000 m
Sample rates, Hz	20, 25, 40, 50, 80, 100, 160, 200, 400, 800
Time synchronization	GPS interface
Temperature stability of the quartz generator	±5 × 10^−9^
Memory	SD card up to 64 Gb

**Table 6:** Main technical characteristics of the self-popup OBSs.

Parameter	Value
GNS	
Sensors	three-component seismometer SM-6, hydrophone HTI-94-SSQ
Natural frequency (SM-6)	4.5 Hz
Sensitivity (SM-6)	28.8 V/(m/s)
Frequency Band (HTI-94-SSQ)	2–30,000 Hz
Sensitivity (HTI-94-SSQ)	12.6 V/Bar (without preamp)
Maximum depth	6000 m
Sample rates, Hz	62.5, 125, 250, 500, 1000, 2000, 4000
Time synchronization	GPS interface
Temperature stability of the quartz generator	± 5 × 10^−9^
Memory	SD card up to 128 Gb
GNS-C	
Sensors	three-component seismometer CME-4111, hydrophone EDBOE RAS
Frequency Band (CME-4111)	0.0083–50 Hz
Sensitivity (CME-4111)	4000 V/(m/s)
Frequency Band (hydrophone)	0.067–30,000 Hz
Sensitivity (hydrophone)	200 V/bar
Maximum depth	6000 m
Sample rates, Hz	62.5, 125, 250, 500, 1000, 2000, 4000
Time synchronization	GPS interface
Temperature stability of the quartz generator	± 5 × 10^−9^
Memory	SD card up to 128 Gb

**Table 7:** Bounds for model parameters used in 4-layer constrained inversion.

Model Parameter	Layer 1 (Sea Water)	Layer 2 (Unfrozen Bottom Sediments)	Layer 3 (IBP/Thawed Sediments)	Layer 4 (Sub-Permafrost Sediments)
Resistivity, Ohm·m	0.25–0.7 (Constrained to within 20% of seawater resistivity estimated from salinity data)	0.8–100	1–200	1–1000
Thickness, m	5–100 (Constrained to within 10% of true model bathymetry value)	5–100	10–200	Not applicable

## Data Availability

Not applicable.
